# Mr. Ring, on Inoculation

**Published:** 1805-03-01

**Authors:** 


### To the Editors of the Medical and Physical Journal.

GENTLEMEN,

IN my last Communication I transmitted some Remarks on a Memoir in the Medical and Chirurgical Review for January; I now beg leave to add some Observations on the remaining part of that article.

We are told, that a case entered on the minutes of Nov. 20, 1804, at the Vaccine Pock Institution, seems worthy of being recorded, as an instance of the various *non-descript* eruptions which occasionally occur during vaccination. After this pompous introduction, which prepares us to expect something extraordinary, who would imagine that this was nothing but a miliary eruption? This eruption is a very common attendant of every febrile disease; it has been *described over and over again;* and is of very little consequence, unless it falls under the observation of an ignorant practitioner, and is mistaken for the small-pox.

The next article in the Memoir, is one relative to the Harrow cases. These, which have caused another false alarm, are nearly akin to those that occurred at Woolwich; and are the natural offspring of the original false alarm at Portsmouth.

By Mr. Bowen's letter to the Committee of the Vaccine Pock Institution, it appears that he vaccinated his child four years and a half ago, when six weeks old; and was satisfied with the result. In order to strengthen the good opinion he entertained of the practice, and to convince Mrs. Bowen of its infallibility, he had inoculated the child every year since with variolous matter; but without the least effect. The last of these trials took place about twelve months ago.

From these repeated proofs, he fully depended on the efficacy of vaccination; but in October last, having a patient in the small-pox, he was induced to inoculate the child once more with variolous matter; and, to his great surprize, a pustule formed on the arm, attended with head-ach, lassitude, fœtid breath, and a pulse upwards of 150. These symptoms were followed by twenty or thirty eruptions; the greater part of which died away in four or five days. Hence it appears, they were miliary eruptions: But there was a pustule on the fore-arm, which proved to be variolous; for, with matter thence obtained, Mr. Bowen

 inoculated inoculated another patient, and produced more than a thousand pustules. Many others were inoculated from this patient, with a similar event; and there is no room to doubt that the disease was the small-pox.

Mr. Bowen had taken the cow-pock matter for his infant from a child of the Rev. Mr. Evans, of Harrow; and, being alarmed by the foregoing result of variolous inoculation in his own child, he mentioned the circumstance to Mrs. Evans, who desired him to put her two children to the same test. These, we are told, seemed to have the symptoms of the small-pox in the same manner with Mr. Bowen's little girl; but in one of them they were more strongly marked than in the other.

There was an eruption, which died away in a few days, leaving only a pustule on the arm, which formed itself fully, and was attended with great inflammation. From this local pustule Mr. Bowen inoculated, and again produced the small-pox.

To this account of the two last cases, a letter from the Rev. Mr. Evans, the father of the children, is subjoined; by which it appears, that in one of them, whose case is particularly described, there was a perfect local variolous pustule, from which matter was taken; and that two children inoculated with it had the small-pox. Two lancets were charged with matter from Mr. Evans's child by gentlemen who went from London. The result of their experiments Mr. Evans had not heard.

It is a melancholy reflection, that when the cow-pock has been publicly known more than six years, the inoculated small-pox more than eighty, and the natural small-pox more than a thousand, many important points relative to those disorders are but little understood by the public in general, nor even by the majority of the medical profession. In the present instances, neither Mr. Bowen, nor the parents of the children knew, that a person who has undergone either the small-pox or the cow-pox is capable of having a local variolous pustule in consequence of inoculation; that this pustule may be accompanied with a rash, or a miliary eruption, or even with constitutional indisposition; and that it is capable of yielding variolous matter fit for inoculation.

The falsehoods and exaggerations that derive their origin from this source, when experiments of the foregoing kind are made, are almost incredible. Even when variolous inoculation totally fails, and the vaccine patient resists the infection of the small-pox, the very experiment gives rise to a variety of false reports.

Several questions were proposed to Mr. Bowen by the Committee of the Vaccine Pock Institution, that have no great tendency to elucidate the point in question; which is, whether the children had the general, or only the local small-pox.

One was, whether the scab left a scar? From this it would seem natural to infer, that if it left a scar, the disease was complete. Another was, whether the child was constitutionally disordered during the *illness*? If there was no constitutional disorder, it cannot well be said there was any illness.

Another question was, "What are the proofs of the child having had the small-pox? In particular, was any child inoculated from him? Here no distinction is made between the inoculation of another subject from the primary or from a secondary pustule; and the answer being given in the affirmative, it was natural for the parents, and even for Mr. Bowen to conclude, that, in the opinion of the Committee of the Vaccine Pock Institution, the child from whom the matter was taken, had the small-pox after the cow-pock. The circumstance of two gentlemen from London taking matter from the pustule on the arm, was sufficient to strengthen that opinion, and to confirm them in their error.

But the principal argument in favor of the opinion entertained by Mr. Bowen and the inhabitants of Harrow, is deduced from the case of Mr. Bowen's child, in whom a secondary pustule appeared. This argument, however, is of but little weight, when we consider how easily this pustule also may have been excited, either by the insertion or contact of variolous matter.

This is a more probable occurrence than some people may suppose; especially in those who are put to the test of a second inoculation; for in such cases there is frequently a considerable degree of itching, which takes place at an early period, and provokes the patient to scratch the arm. Hence a secondary pustule is a common occurrence, occasioned by a secondary inoculation with the nail.

I am far from denying the possibility of a pustulous eruption of the variolous kind, in those who have already had the small-pox or the cow-pox; but it is not a common event. When it happens after variolous inoculation, a number of pustules break out round the primary pustule, and and form a cluster, which becomes more or less confluent; whereas in Mr. Bowen's child, there was only one solitary pustule on the fore-arm.

It has been very properly remarked, that ere vaccination is condemned, experiments ought to be made before witnesses; and not in a corner. I cannot help thinking also, that they ought to be made before impartial and *disinterested* witnessess, which medical practitioners can never be. I am far from concluding, that Mr. Bowen was influenced in his decision by any personal consideration; but at the same time it is only justice to mention, that in consequence of this alarm, he has considerably extended his practice, and inoculated nearly two hundred persons with the small-pox; spreading that dreadful disease from Harrow to the metropolis.

Could it be proved, that variolous inoculation will now and then produce a secondary variolous pustule in those who have undergone vaccination, it is of very little consequence, when we consider that the operation is optional, and no longer necessary to be performed.

The next article in the Memoir is the following, communicated by Mr. Freake, from his notes, taken at the time when the cases occurred.

On July the 8th, 1804, he was sent for to attend a child of Mr. Waters, milkman, of Kentish Town, who was dangerously ill of convulsions, of which it died. It had been attacked three days before with the symptoms that usually precede the small-pox. On the 18th of the same month, another child sickened with the small-pox of the confluent kind; from which it recovered with difficulty. On the 3d of August, a third child of the same family sickened with the small-pox, which proved distinct, and terminated favourably.

These three children had been inoculated by Mr. Wetherall, of Highgate, about eighteen months before; who, on inquiry, declared they were inoculated with variolous matter; that they all had the eruptive fever, and a fair shew of pustules.

What will our alarmists say to this? Possibly they may tells us, these children were inoculated with the chicken-pox matter. Admitting this to be the case, it is no argument in favour of variolous inoculation, which gives rise to a much greater number of mistakes, and a much greater number of failures than the inoculation of the cow-pock. The next article is a communication from Dr. Bremer of Berlin, who gives an account of a hundred thousand cases of of vaccination, which have come more or less under his cognizance, without a single instance of subsequent small-pox. Dr. Bremer has made some experiments with equine matter, sent to him from Vienna and Milan; and produced a disease resembling the genuine cow-pock to the fourth generation; nevertheless, it seems, he will not confide in it, nor admit the theory which has been offered on the subject. This shews, that prejudice and obstinacy are not entirely confined to the enemies of vaccination.

With respect to Dr. De Carro's letter, an extract of which follows in the same Memoir, I agree with that gentleman, that if the experiments of Drs. Loy, Sacco, and La Font, have not convinced certain sceptics of the origin of the cow-pock, nothing can. Dr. De Carro remarks, as I long ago observed in my Treatise on the Cow-pox, that the proofs in favour of Dr. Jenner's opinion are positive, the proofs to the contrary are negative. Dr. De Carro thinks his correspondent too good a logician not to perceive the difference between such arguments; but we must once more recollect, that there are none so blind as those who will not see.

Experiments made by Dr. Loy and Dr. Sacco in public, in the presence of proper judges and respectable witnesses, cannot fail to convert every candid and unprejudiced opponent. Nevertheless, even in the last number of the Critical Review we meet with one writer, who affects to doubt, or rather to disbelieve this opinion; but, whoever he may be, he can have very little claim to the character of a logician.

That writer enters into a controversy with Dr. Squirrel, respecting the comparative merits of physicians and apothecaries in inoculation. It should, however, be recollected, that there is a third description of medical men, who have a considerable share in inoculation, and to whom it is not a little indebted. It may, perhaps, be difficult to name one considerable improvement in this branch of practice, introduced by any practitioner who was not educated a surgeon.

By this observation, no disrespect is intended to be shewn towards those gentlemen who are educated as physicians. On the contrary, it is with me a subject of deep regret, that men of their talents do not more generally condescend to cultivate the practice.

The next passage in Dr. De Carro's letter is curious and interesting. He says, an English gentleman, Mr. Hamilton, who was Secretary to Lord Elgin, told me, that in reading reading M. Auban's report of the cow-pock found in two villages near Constantinople, he had been much struck by the circumstance, either unknown to M. Auban, or not mentioned by him, that these two villages are precisely the place, where seven or eight hundred horses, from the stables of the Grand Seignor, are sent out to graze every year. Is it not very likely, that it is the grease of these horses, which has rendered the cow-pock endemic in those villages?

Dr. De Carro disapproves of a violent style in literary discussions; and perhaps, there is no style more objectionable, except the style of adulation. From the number of answers which have appeared, he thinks Mr. Goldson's pamphlet has caused much mischief in England. It is, however, the decided opinion of most people, and particularly of our most eminent Reviewers, that in consequence of the answers he has called forth, there are few men in England to whom vaccination is more indebted than Mr. Goldson.

Dr. De Carro observes, that matter procured by triturating and dissolving the vaccine crust generally proves successful, particularly in the practice of Dr. Valentin, of Nancy. I have lately received from that learned and enlightened physician and philanthropist, who is now removed to Marseilles on account of his health, two letters; in one of which he informs me, that he had successfully vaccinated in this manner twenty-four persons; among the rest an infant seventeen hours after its birth, whose health did not appear to be in the least affected thereby. In this case the crust was seventy-two days old, in others eighty-three days; yet the vesicle which ensued was as perfect, as if it had been produced by fluid matter taken immediately from the arm.

In the other letter he desires me to acquaint Dr. Jenner, that a considerable number of inoculators, in Paris and the provinces of France, have vaccinated children affected with herpetic eruptions, the scald head, crusta lactea, and other cutaneous diseases, without observing the degeneration of the vaccine vesicles of which Dr. Jenner speaks; that the cow-pock matter taken from those vesicles produced the cow-pock in other subjects; and that both descriptions of patients were preserved from the small-pox. These experiments were repeated, particularly in the hospitals at Paris, in hundreds of individuals; and always with the same result. Hence they conclude, that there must must be some extraordinary peculiarities, unknown to them, in the subjects of whom Dr. Jenner speaks.

This is a very valuable testimonial in favour of vaccination, since it proves that it is much less fallacious than might have been apprehended from the experience of Dr. Jenner in Gloucestershire. I have vaccinated numbers of children labouring under such disorders; and have seldom been frustrated in my endeavours to excite the genuine disease.

I therefore congratulate Dr. Jenner and the public on this occasion. Mr. Goldson congratulates himself on having given rise to Dr. Jenner's paper in the Journal. It will be seen, upon examination, that the subject is not altogether new; but Dr. Jenner has here enlarged upon it. Whatever may be its value or importance, it reflects very little honour on Mr. Goldson; for if it was occasioned by his pamphlet, it is only an instance of good springing out of evil.

This is not the first instance on record in the annals of vaccination, nor will it be the last. One has just occurred at Clapham, where, from well-known causes, the practice has not hitherto been very popular. I was lately informed that the small-pox raged in the village, where it was introduced by one of the inhabitants, who had been inoculated at the Small-pox Hospital, and that several persons had fallen victims to the disorder. I therefore wrote to Mr. Gardner, a very respectable surgeon of that place, who, at my request, had undertaken to vaccinate gratuitously under the sanction of the Royal Jennerian Society, desiring to know whether the report was true, and what success he had met with in vaccination.

His reply was, that from all he could learn, the child by whom the small-pox was introduced into Clapham had been inoculated at the Small-pox Hospital; and many others had been inoculated at the same place. The number whom Mr. Gardner had vaccinated gratuitously was but small; owing to a particular shyness in the people of that neighbourhood to submit to vaccination; but the fatality that had just happened there from the small-pox had alarmed them; and they were then beginning to come forward. Mr. Gardner likewise informed me, that all those vaccinated by him have gone on extremely well; and that I might depend on his exertions.

He was faithful to his promise; and I have great reason to congratulate him and myself on his success; for I received another letter from him five days afterwards, with the the happy intelligence, that since writing before, he had vaccinated about fifty poor people. Many, however, still declined his offer, because they can so easily be inoculated at the Hospital with what they call the real small-pox. Several other patients from Clapham had within a few days imported fresh cargoes of variolous infection from the same quarter; and those who were previously inoculated there, still laboured under the disease.

Happily, the quarantine laws are now under revision; and it is hoped that a power will be vested in government, if no power is already vested, to subject persons who carry variolous infection about them to those laws. It is also necessary to prohibit the inoculation of that disease, which is now the greatest obstacle to vaccination. Another desirable object is, to settle upon every Inoculator of the Small-pox, and every person connected with institutions for that purpose, a pension for life, equal to the professional emolument he may now receive. This would, perhaps, enable gentlemen to see reasons for discontinuing the inoculation of the small-pox.

The next article in the Review is a Resolution entered into by several gentlemen of the Faculty at Plymouth, "*not to inoculate for the small-pox, excepting after the inoculation for the cow-pox, and that only in peculiar circumstances, where an experiment may be required to satisfy doubting parents.*"

This is precisely what I have long professed, and the opinion I published, which drew on me so much censure and insult. But when I recollect the quarters from which it came, and the modes in which articles, written by an envious opponent, find their way into a Review, I can look on such attacks with proper contempt.

Having shewn that the test of variolous inoculation was not void of danger, and therefore not to be employed wantonly, a writer in the Monthly Review maintains that, according to this doctrine, we are not justifiable in putting children to this test, even at the desire of their parents.

Stulti, dum vitant vitia, in contraria currunt.

Of evils, we are to choose the least; and it is certainly a much smaller evil to vaccinate and variolate every human being, than trust them to the small-pox even under inoculation: But the writer alluded to is guilty of a gross and palpable perversion of truth, when he asserts that there is no danger in the experiment. When I consider such experiments disgraceful, it is because I consider them unnecessary, unnecessary. It was unnecessary to be alarmed by such cases as Mr. Goldson's; and if any persons were alarmed by them, it was owing to their ignorance of the subject. If they were not alarmed, such experiments could not have been instituted by them with any other view but that of ostentation, and rendering themselves conspicuous.—Medical practitioners always increase a panic when they appear to feel it themselves.

The Reviewer says, I assign no other reason for calling the practice disgraceful, than that I deem it unnecessary; yet in the next page he admits, I have brought evidence of its having proved fatal. This, he says, happened forty years ago; and not since the introduction of the cow-pox. A case, however, has lately happened in the family of Dr. Stewart, of Plymouth, which occasioned a serious alarm; and, together with other circumstances, induced the gentlemen of Plymouth to adopt my opinion.

The Reviewer (as well as the Reviewer in your own Journal) is inaccurate in considering such occurrences rare; I have mentioned several in my Treatise on the Cow-pox, and in my Answer to Mr. Goldson; and Buchan, in his Treatise on the Small-pox, has also the following case. "I have known a nurse, who had had the small-pox before, so infected by lying constantly a bed with a child in a bad kind of small-pox, that she had not only a great number of pustules, which broke out all over her body, but afterwards a malignant fever, which terminated in a great number of imposthumes or boils, and from which she narrowly escaped with her life. I mention this to put others upon their guard against the danger of this virulent infection."

The writer in the Monthly Review afterwards affirms, that I allow the insertion of variolous matter to be a more complete test than exposure to the natural infection. This is another misrepresentation. The reason why I call the evidence there adduced still more convincing is, that some of the cases were of longer standing when the test was employed; not that the test of inoculation is more decisive than that of the natural infection. On the contrary, Mr. Goldson and others contend, that inoculation with variolous matter is not the strongest test. He should, however, have recollected, that the vaccine patients put to that test at the Small-pox Hospital, were exposed to the full infection of the small-pox, in the way of effluvia at the same time; and even shook hands with those who laboured under der the disease. This has also been done in every part of the metropolis with impunity.

The Reviewer accuses me of unequivocally condemning a farther examination of the preventive powers of the cow-pock. This is a false accusation, and must be known to be such, to all who have read my pamphlet with proper attention. I encouraged the test of inoculation with variolous matter till the public had confidence in the practice; I still encouraged the test of exposure to natural infection; which I considered more safe to the patient, and equally satisfactory to others. It is probable, no practitioner has so strenuously recommended this test, and few have had so many patients to whom they could recommend it. A writer in one Review, who also espouses the part of Mr. Goldson, gravely asserts, that if Mr. Dunning had declined putting his patients to the test of variolation it might have been excuseable, because he had so often put them to the test of the natural infection; for me, who had much oftener put my patients to the same test, no excuse was to be admitted.

Such reflections from such quarters I can easily bear, while I have the consolation to find, that some of those who were foremost in submitting their vaccine patients to the touchstone of variolous inoculation are now sensible of their error; and come forward to caution others against the same hazardous attempt.

The next article in the Medical and Chirurgical Review contains some opinions that are well-founded, and may be considered as genuine; others that are ill-founded, and may be considered as spurious. Among the first we may reckon the opinion, that a second, or if necessary, a third inoculation of the cow-pock, is a good counter-proof; among the second we may reckon the opinion, that the cow-pock cannot occur a second time.

In the next article we are informed, Mr. Goldson has ascertained by experiment, that the cow-pock produced by inoculation in the hand is blue; this is what I told that gentleman long ago. It seems, however, still necessary to tell him, that a blue cow-pock yields the same matter as a cow-pock of any other colour. The quality of the matter is no more altered from its being contained in a blue pustule, than the quality of wine from its being contained in a blue decanter.

An article has at length found its way into the aforesaid Review, that was formerly inserted in this, and in the Medical Review. It is said to come from Prof. Nessi, of Pavia.

Pavia. It asserts the antiquity of the cow-pock, on the authority of Marias, first bishop of Lausanne. I long ago pointed out this error; and remarked, that the disease alluded to is the murrain. This error has also crept into the Bibliotheque Britannique.

While the failures, or apparent failures, of vaccine inoculation are resounded on every side, it would be a dereliction of duty not to enumerate some of the failures, or apparent failures, of variolous inoculation. I shall therefore occasionally make an addition to the catalogue I have already commenced.

1. Mr. Wood, of the Swan, in Portland Street, was nursed for the small-pox when a child. He has lately had the disorder in rather a severe degree.

2. In my last I mentioned the case of Mrs. Smith of Kensington. Another young woman was inoculated with her, from the same subject; and was supposed to have the disease in a regular manner. Two years after, she went to see her sister, who was recovering from the small pox; and, conceiving there was no danger, slept in the sheets which had been used by her sister; in consequence of which she caught the small-pox and died.

3. Mr. Geary, No. 7, Dean Street, Soho, was inoculated for the small-pox when seven years old, and apparently had the disorder, attended with great constitutional indisposition. He had had the chicken-pox in his infancy, otherwise this affection might have been supposed to be mistaken for the small-pox. When ten years old, he caught the small-pox, and had a very heavy burden, leaving considerable marks behind.

4. The case of Mr. Langford, related in vol. 4, of the Memoirs of the Medical Society, has been doubted by some people; but no case on record, of any description, is better authenticated. I know different persons who are well acquainted with the fact. Mrs. Cook, of Kemp's Court, Berwick Street, knew him well, and declares that he was much pitted by the first attack of the distemper; of the second he died.

5. Mr. O'Keeffe's child, No. 58, Lower Sloane Street, was inoculated for the small-pox. A pustule rose, and the medical practitioner who inoculated her declared her to be safe. Two years after, she had the disease in the natural way very severely.

6. The father of Mrs. Barlowe, No. 44, in the same street, was attended for the small-pox when young. He afterwards caught it from his children, had a heavy load, and lost an eye by the disorder.

7. A child of a gentleman at No. 40, in the same street, after being inoculated for the small-pox, and declared safe, also had the disease.

8. A relation of an eminent physician, who was inoculated by the late Dr. Glass, of Exeter, and declared safe, afterwards had the same disease in the natural way.

9. A son of Mr. Giles, No. 6, Water-lane, Blackfriars, was inoculated for the small-pox. His arm rose, and an eruption took place, with which the medical practitioner and the parents were satisfied. Four years after this, he died of the small-pox.

10. Mr. Chamberlaine, surgeon, of Clerkenwell, was attended in a disease which was declared to be the small-pox by two eminent physicians; he since had that affection in a most unquestionable manner.

11. A similar instance occurred in an aunt of Mr. Hurlock, surgeon, of St. Paul's Church-yard. The first and second attack were such, as to leave no room for doubt that she really had the small-pox twice.

12. A child of Mr. Britain, in Little Portland-street, was inoculated for the small-pox by a respectable practitioner, and considered safe; but has since had the disease.

13. The case related in the Medical Journal for May, 1801, by Mr. Purton, of Alcester, is incontrovertible.

14 Another case is there alluded to, which Mr. Purton says was well authenticated, and sufficient to stagger the firmest sceptic.

15. Mrs. Chamberlaine, No. 14, Maiden-lane, Wood-street, had the confluent small-pox when a year old; but whilst nursing one of her children, she sickened, and had about twenty pustules. Her constitutional indisposition was so considerable, that she afterwards went through a course of physic. She since nursed another of her children in the small-pox, and a similar eruption, together with similar constitutional symptoms, occurred; on which account she submitted to another course of physic.

16. One of her daughters, when a year old, slept in the same room with another child who died of the small-pox. She sickened, and had an eruption, which the medical gentleman attending her supposed to be the same disorder. A year after she caught the disorder, and had a heavy burden.

These cases will suffice for the present; others shall appear in due time, if occasion should require. They are the best answer to those who look for infallibility in any practitioner, or any practice.

I am,

I am now favoured with a very valuable and interesting work, entitled, A Report on the Progress of Vaccine Inoculation in Bengal; by John Shoolbred, Superintendant-General of Vaccine Inoculation. This work was printed at Calcutta; and has been reprinted in London.

The following is an Extract from the proceedings of the Governor General in Council, dated May 3, 1804.

"Ordered, That the Report on the Progress of Vaccination in Bengal, and in the provinces subject to the immediate authority of this Government, from the period of its introduction in 1802, to the end of the year 1803, submitted to the Medical Board at Fort William, by Mr. J. Shoolbred, Superintendant-General of Vaccine Inoculation, be published for general information, at the expence of the Hon. Company; and that Mr. Shoolbred be desired to superintend the publication.

"The Governor General entertains a just sense of the zeal, diligence, and ability manifested by Mr. Shoolbred, in the discharge of the important duty committed to him, as Superintendant-General of Vaccination. The Report submitted to Government by Mr. Shoolbred, affords abundant evidence of the difficulties opposed to the preservation and extension of the benefits of Dr. Jenner's discovery in this country; as well as of the indefatigable assiduity and public spirit, with which every obstacle to the success of the orders of Government has been encountered and surmounted by Mr. Shoolbred, and by his predecessor, Mr. William Russell.

"The support and assistance which Mr. Shoolbred has received from the medical profession, and from others residing as well at Calcutta as in the distant provinces, are highly creditable to the gentlemen whose exertions are noticed in Mr. Shoolbred's Report; and the success with which those laudable and disinterested exertions have already been attended, affords a reasonable ground of expectation, that the invaluable benefits of Dr. Jenner's discovery will be preserved in perpetuity in these extensive and populous provinces; and that they will in time be disseminated through every part of Asia."

[To be continued.]

